# Technology-Enhanced Peer Support for Depression in Older Adults: Single-Arm Mixed Methods Feasibility Study

**DOI:** 10.2196/85526

**Published:** 2026-07-07

**Authors:** Jin hui Joo, Alice Xie, Samuel Van Vleet, Joseph Locascio, Kristina Brackpool, Fei Wu, Namkee Choi, Phyllis Solomon, Nicholas Cloney

**Affiliations:** 1 Department of Psychiatry Harvard Medical School Massachusetts General Hospital Boston, MA United States; 2 Department of Neurology Harvard Catalyst Biostatistical Group Massachusetts General Hospital Boston, MA United States; 3 Department of Global Health and Population Harvard T. H. Chan School of Public Health Harvard University Boston, MA United States; 4 School of Social Work The University of Texas at Austin Austin, TX United States; 5 School of Social Policy & Practice University of Pennsylvania Philadelphia, PA United States; 6 Brigham and Women's Hospital Boston, MA United States

**Keywords:** aging, depression, health services research, intervention, mental health, older adults, peer support, psychosocial, social support

## Abstract

**Background:**

Depression in late life is often compounded by social isolation and barriers to care. There is limited study of technology-enhanced peer support for depression among older adults.

**Objective:**

This study aimed to assess the feasibility and acceptability of a technology-enhanced peer support intervention to decrease depression among older adults.

**Methods:**

We used a mixed methods pilot study among adults aged 50 years and older with depression who received a peer support intervention called Peers+. The intervention consisted of 8 weekly video chats and unidirectional texts focused on increasing depression self-care and coping. Data obtained from screening, baseline, postintervention, and 3-month follow-up were used in the analysis to assess preliminary outcomes of the intervention. Mixed effects longitudinal models were used to assess change in depression, and qualitative data were collected and analyzed to identify key themes related to participant experiences.

**Results:**

A total of 34 older adults with a mean age of 67 (SD 9.57) years participated in the study, and 82.4% (28/34) of participants finished all 8 intervention meetings. Depressive symptoms declined over the course of the study of 35 weeks (*F*_1, 88.8_=26.0; *β*=–.14, 95% CI –0.20 to 0.09; *P*<.001). Emotional well-being (*β*=.48, 95% CI 0.26-0.70; *P*<.001), social functioning (*β*=.71, 95% CI 0.33-1.09; *P*<.001), self-efficacy (*β*=2.29, 95% CI 0.83-3.75; *P*<.001), and coping (*β*=2.90, 95% CI 0.24-5.55; *P*<.001) improved throughout the study period. Participants perceived supportive texts as reinforcing trust between peer coaches, using coping strategies, increasing social connection, and providing accountability for improving self-care. Peer coaches and older adults needed technology support for participation in the study.

**Conclusions:**

This study demonstrated the feasibility and acceptability of a peer support intervention enhanced by video chats and texts, delivered by older adult peer coaches to an ethnically diverse group of older adults with depression. Study findings indicate that ongoing and accessible technology support contributed to older adult participation and engagement.

## Introduction

### Overview

Depression affects up to 15% of older adults and is a leading contributor to the overall burden of disease among older adults worldwide [1]. The number of older adults with depressive symptoms is estimated to increase from 3.8 million to 8.2 million between 2005 and 2060 [2,3]. Risk factors for depression include loneliness, social isolation, low socioeconomic status, and education, disproportionately affecting people of color [4-6]. Untreated depression is associated with poorer physical functioning, various cooccurring mental health disorders, cognitive impairment, lower quality of life, and increased risk of suicide [7-10]. Although evidence-based treatments exist, access and engagement remain low because of stigma, limited awareness of treatment options, financial barriers, and limited-service capacity [11]. More than half of older adults in the United States with indicated need do not access care for depression [12]. In addition to intractable barriers to access, there are an estimated 1.2 million behavioral health providers to treat 59.2 million people who seek mental health services [13,14]. For the geriatric population, the United States faces a significant shortage of mental health providers, which poses a serious challenge to meeting the mental health needs of an increasingly aging population [15].

To address these challenges, we used task shifting, defined as a redistribution of selected tasks from specialized clinicians to trained lay health workers who receive focused training on how to perform a limited set of tasks [16,17]. Task shifting aspects of depression care by training peer support workers to provide social support and encourage self-care may effectively engage older adults, reduce stigma, and increase access with a positive impact on depression outcomes while mitigating workforce challenges.

Peer support involves connecting a person who has a health problem with someone (a peer) who has a lived experience of the same health condition, someone who “has been there” and “lived it” [18-21]. The practice of peer support is most common for specific populations, such as those with persistent mental illness and substance abuse, although studies have investigated its use in a range of medical and psychiatric conditions [22,23]. Two meta-analyses have shown that peer interventions can decrease depressive symptoms with moderate effect sizes in adult populations [24,25]; however, peer-led groups were more commonly used rather than individual peer support, which may be more engaging for older adults due to the stigma of seeking help for mental health. Studies of peer support also often lack intervention manualization, use of fidelity measures, and understanding of the underlying mechanisms [26-28]. While peer support is widely practiced, it has not been rigorously studied for use among older adults, and the integration of technology with peer support to enhance accessibility and reach is limited [29].

Technology-based health service delivery has become increasingly common, and there has been a shift toward telehealth adoption since the COVID-19 pandemic. Telehealth for depression has been shown to be highly acceptable and effective, and many older adults who lack transportation or experience mobility restrictions report a preference for it [30,31]. Smartphones are now used by more than half of older adults, and both smartphones and text messaging have emerged as potentially effective ways of delivering mental health support to this group [32,33]. A systematic review of randomized controlled trials (RCTs) including 764 adults diagnosed with depression found that interventions delivering 1-5 daily text messages were effective for improving depressive symptoms; however, effect sizes have varied considerably across studies [34]. Although rare, interventions that integrate texting or use it as the primary intervention for older adults have demonstrated a decrease in depression, and texting may be used to deliver social support and learning of coping skills [35,36].

### Peers+: Conceptual Framework

The Peers+ intervention is informed by theories of social learning, social support, and principles of behavior change. Older adults who have lived experience of depression and a knowledge base of depression self-care skills and shared cultural experiences are selected, trained, and supervised to engage the depressed older adult in a trusting relationship in which social learning occurs. The shared experience of depression and age between the peer coach (PC) and participant provides a strong foundation and a sense of shared identity that contributes to a strong working alliance [37]. Within the context of a strong working alliance (bonded relationship and agreement on goals), the peer provides aspects of social support, specifically, emotional, appraisal, and informational support that buffers stress and provides strategies to reduce depression [38]. The PC uses social persuasion, modeling, and encouragement so that people can experience success in coping with depression and develop a stronger belief in their own capabilities [39]. The result is increased self-efficacy and a sense of empowerment to continue self-care independently [40].

### This Study

In this study, our goal was to assess the feasibility and acceptability of a technology-enhanced peer support intervention (Peers+) delivered by older adult PCs to decrease depression among older adult participants. Peers+ was adapted from a peer support intervention for depressed older adults that was delivered by telephone and tested in an RCT during the COVID-19 pandemic [41]. In this study, we used a mixed methods approach to assess preliminary outcomes and to understand participant and PC experiences of the intervention, specifically the use of video chats and supportive texts to reinforce engagement and increase coping. Our primary aim was to evaluate the feasibility and acceptability of Peers+ and assess intervention fidelity. Secondary aims were to examine preliminary changes in clinical and psychosocial outcomes, including depressive symptoms (primary outcome), anxiety, loneliness, coping, and self-efficacy. We hypothesized that Peers+ would meet feasibility and acceptability benchmarks and that participation would be associated with reductions in depressive symptoms and improvements in the secondary outcomes.

## Methods

### Study Design and Procedures

Peers+ was an open pilot study using a sequential explanatory mixed methods design. Quantitative data were collected and analyzed first, with qualitative data collected in a second phase to explain and enrich the quantitative results. Integration occurred during interpretation: thematic findings were used to clarify outcome trajectories, identify perceived mechanisms of change, and characterize contextual factors shaping engagement and participant experience. More details on this process can be found in the respective sections regarding the quantitative and qualitative analysis methods. Quantitative data were collected at baseline, postintervention, and 3 months from December 2022 to January 2025. The benchmark for feasibility was set as ≥80% of participants who complete at least 80% of meetings with the PC, and acceptability was assessed with postintervention semistructured interviews that probed participant experiences. Preliminary outcomes included depression, well-being, coping, self-efficacy, and social functioning. The study was approved by the Massachusetts General Brigham Institutional Review Board (National Clinical Trial number 05611996).

### Older Adult Participants

Participants were recruited through community outreach, newspapers, and online advertisements. Study staff screened candidates by telephone for eligibility criteria that included the following: (1) aged 50 years and older, (2) depressive symptoms (Patient Health Questionnaire-9 [PHQ-9] scores ≥10), and (3) ability to give informed consent. Exclusion criteria were: (1) current mania or hypomania, (2) psychotic syndrome, (3) substance abuse or dependence in the past 12 months, (4) acute suicidal ideation, (5) receiving psychotherapy more than once a month, (6) history of dosage change in psychotropic medication in the past 3 months, and (7) lack of access to a device that could receive text messages. Psychiatric symptoms were assessed with the Mini-International Neuropsychiatric Interview [42].

Eligible candidates were assessed by a mental health professional who conducted a brief clinical assessment to understand the psychosocial factors contributing to the participant’s depression. Those who were eligible were guided through an informed consent process. After consent was obtained, participants underwent a baseline assessment, after which participants were matched with a PC who contacted the participant and scheduled the first meeting. Study staff provided instructions on accessing Zoom (Zoom Communications, Inc) for video chats and texts on their mobile telephones. To increase access to the study, participants were allowed to use the telephone dial-in function on the Zoom platform.

### Intervention Contents and Procedures

Peers+ is delivered by older adult PCs with lived experience of depression. The intervention consists of 8 weekly video chats lasting 45-60 minutes with a PC and includes the following three components: (1) relationship building, active listening, emotionally responsive, person-centered communication, and discussion of stressors; (2) goal setting, brainstorming and discussion of coping strategies, and modeling and communication of motivational messages regarding self-care; and (3) informational support and encouragement to use community and clinical resources.

In addition to video chats, 3-5 supportive texts between weekly meetings, starting from the second week, are sent by the PC. The texts are designed as simple, unidirectional enhancements to the intervention to promote coping and enhance social connection in the context of a trusting relationship that has already been established. The messages are personalized, containing relevant affirmations (eg, “you are doing a great job”) and coping skills (eg, “take time out for yourself”) and are sent using a simple text that is accessible via a cellular telephone.

### Training and Supervision of PCs

PCs who delivered the intervention were recruited from the community and peer support networks through word of mouth and referrals from peer support organizations. After undergoing an interview with the study team, interested candidates who met the following criteria were invited to participate: (1) aged 50 years and older, (2) a history of depression, (3) self-report of at least 5 years in recovery and the ability to perform the peer role in the study, and (4) prior work or volunteer experience in mental health. Eligible candidates then underwent informed consent and were invited to participate in training. A total of 6 PCs were trained. One PC left the study for permanent employment.

The study principal investigator (JhJ) and study staff conducted 20 hours of training that included topics such as using a relationship-centered approach, effective communication, appropriate self-disclosure, establishing boundaries, participant and peer safety and confidentiality, expressing empathy, active listening, goal setting, and sharing coping skills. Training activities featured didactic presentations, role-play, and case discussions. PCs received a total of 5 hours of training on using Zoom and Voice over IP SMS platforms for video chats and texting. PCs, with the assistance of study staff, were also trained to create personalized text messages based on discussions during supervision meetings. When PCs were matched and meeting with participants, they attended weekly supervision meetings with the mental health professional, where they discussed progress and problem-solved challenges, with the meetings serving to develop PC skills and provide quality assurance.

### Fidelity

Each participant meeting with the PC was audio recorded on Zoom. Study staff reviewed the audio recordings and rated them using a fidelity checklist based on five criteria: (1) listens actively, (2) uses person-centered communication, (3) uses emotionally responsive communication, (4) works toward self-care and improved coping, and (5) supports autonomy and independence. Each criterion was rated from 0 to 4 for a total score of 0-20. For the first match, the study staff reviewed audio recordings from the first, fourth, and eighth meetings. For all subsequent matches, 2 audio recordings between meetings 2 and 7 were randomly chosen and reviewed. For the text message component, a study staff member reviewed the frequency and content of the text messages weekly for each PC-participant dyad for quality assurance and fidelity.

### Safety

To assess and manage suicide risk, established protocols for suicidality were followed throughout the study. After initial screening, a mental health professional assessed the older adults for exclusion criteria, including suicidal ideation. PCs and study staff were trained to alert the mental health professional if the participant expressed suicidal ideation, and the participant was assessed and referred to emergency psychiatric care as needed.

### Data Collection

Demographic information such as age, gender, ethnicity, and education was collected. We measured engagement as ≥80% attendance (at least 6 weekly meetings). Medical comorbidity was measured with the Composite International Diagnostic Interview found in the World Health Organization’s World Mental Health 2000 Chronic Physical Conditions [43].

### Preliminary Outcome Measures

We used validated measures that have been used in older adult populations to assess changes across time in the outcomes of interest. Depression was measured with the 9-item PHQ-9 [44]. Each item is scored on a 4-point Likert scale, with scores ranging from 0 to 27. Higher scores indicate higher levels of depression. Additional variables of interest included self-efficacy, coping, emotional well-being, and social functioning. Self-efficacy was measured with the General Self-Efficacy Scale [45]. The General Self-Efficacy Scale is composed of 10 items, and each is scored on a 4-point Likert scale. Scores range from 10 to 40, with higher scores indicating greater self-efficacy. Social functioning and emotional well-being were assessed using subscales of the Medical Outcomes Study 36-Item Short Form Health Survey, a widely used self-report survey that measures health-related quality of life [46]. Higher scores indicate better social functioning and emotional well-being. Adaptive coping was assessed through the Brief Coping Orientation to Problems Experienced survey [47]. The scale ranges from 1-4 and contains 3 subscales: problem-focused coping, emotion-focused coping, and avoidant coping. Adaptive coping was assessed by combining problem-focused and emotion-focused strategies. Higher scores indicate better adaptive coping.

### Semistructured Interviews

Trained study staff conducted semistructured interviews with participants and PCs upon completion of the intervention on Zoom. Each interview lasted 30-45 minutes. Questions posed to participants included perceived strengths and weaknesses of the intervention, their relationship with the PC, and what they gained from participation and experience with technology. PCs were asked questions regarding training, supervision, perceived strengths and weaknesses of the intervention, and experience with using video chats and texting for the intervention. Interviews were audio-recorded and professionally transcribed using a transcription service.

### Sample Size and Statistical Analysis

A power analysis was not performed. Pilot studies inherently involve small sample sizes so that any estimates of parameters (eg, effect sizes, SDs, and recruitment rates) can be imprecise and variable. All enrolled participants were retained in the analyses even if one or more assessments were missing. For each outcome, we used all available observations across time for each participant via maximum likelihood estimation under the missing at random assumption. No imputations across different outcomes were performed. Assessments were measured at baseline, 8 weeks, and 3 months after completion of the intervention. The maximum duration in the study was up to 35 weeks, associated with rescheduling of meetings, physical illness, medical appointments, and other life events.

Baseline characteristics and clinical and social outcomes were summarized. We conducted mixed effects longitudinal analyses to predict 5 dependent variables (depression, self-efficacy, social functioning, adaptive coping, and emotional well-being) in separate analyses, with fixed effects predictors of numeric time (measured in weeks from baseline with linear and quadratic terms), as well as age, years of education, and their interactions with time. Random effects included participant-specific intercepts and slopes for linear time. A Kenward-Roger adjustment for degrees of freedom was used. Higher-order fixed and random terms were initially tested but removed if nonsignificant to improve model parsimony and statistical power. Adjusted means, partial regression coefficients, and proportion of variance accounted for by model fixed effects in each outcome were used as indices of effect size. Model residuals were examined graphically to confirm reasonable adherence to assumptions of normality. Additionally, we examined possible differences in depressive symptoms based on those who used Zoom vs phone calls through a mixed effects longitudinal model. The analysis included time (both linear and quadratic), method of contact (Zoom vs phone), their interaction, and demographic covariates such as age and education. Random effects accounted for individual differences in symptom trajectories. For all analyses, a significance level of α=.05 was used for 2-sided tests unless otherwise specified. These analyses and data visualizations were conducted using SAS software (version 9.4; SAS Institute Inc;).

### Qualitative Data Analysis

Transcripts from the postintervention semistructured interviews were analyzed using thematic analysis, a rigorous and systematic qualitative method for identifying, analyzing, and reporting patterns of meaning across a dataset [48]. Three study team members worked collaboratively across all phases of analysis. The analytic process followed an iterative, inductive, multistage approach. In the first stage, 3 study team members independently reviewed the same initial set of 3 transcripts to develop familiarity with the data and generate a preliminary codebook. Inductive coding was prioritized to allow themes to emerge directly from the data rather than being imposed by a predetermined conceptual framework. After an independent review, team members met to compare and discuss the preliminary codes, reconciling differences in interpretation and arriving at a consensus for each code. The initial codebook was used for subsequent transcripts, and any new codes were added to the codebook. Once all transcripts were coded, the team members moved to theme generation. During this phase, the full set of codes was reviewed, patterns were identified, and codes were grouped into themes that were refined through discussion [49]. Final themes were reviewed against the original transcripts to ensure they accurately represented the data and were grounded in participant accounts. All analyses were conducted using Dedoose (version 10.0.59; Socio Cultural Research Consultants, LLC), a web-based platform designed to support mixed methods and qualitative data management and analysis.

### Ethical Considerations

This study was approved by the Massachusetts General Brigham Institutional Review Board (2022A008289). Approval was granted in June 2020. All participants provided oral informed consent prior to any study procedures. Consent materials were available in English, and interpreters were available upon request. Participant privacy and data security were protected through multiple safeguards. Study data were collected and stored in REDCap (Research Electronic Data Capture; Vanderbilt University) on institutional servers, with access limited to authorized study staff. All data were coded with a unique study ID and stored separately from identifiers. The data were anonymized. Files were stored on encrypted, access-controlled servers, with 2-factor authentication required. Synchronous contacts were conducted via Zoom, and supportive texts were delivered via Google Voice. PCs received training on confidentiality and privacy protections. Participants received US $30 per assessment and interview, provided as gift cards or checks.

## Results

### Participant Characteristics

Participant characteristics (N=34) are summarized in Table 1. The mean age of participants was 66.7 (SD 9.57) years. The majority of the participants were female (31/34, 91.2%). Slightly more than half of the participants identified as White (19/34, 55.9%), and the remainder of the sample was represented by Black (8/34, 23.5%), multiracial (5/34, 14.7%), Asian (2/34, 5.9%), and Hispanic (1/34, 2.9%) participants. Greater than 50% (21/34) completed a college degree, and approximately 25% (8/34) had attended community college or technical school. The majority (21/34, 61.8%) lived alone and had a history of counseling (26/34, 76.5%). At baseline, the mean depression score measured by the PHQ-9 was 11.32 (SD 5.36).

The mean age of PCs was 59.8 (SD 8.0) years, ranging from 50.0 to 73.0 years. All PCs were female (6/6, 100%), and two-thirds were White and one-third Black. One-half of the PCs had a college degree, with 1 having an Associate’s degree and 2 with graduate or professional degrees. Two-thirds of PCs were currently involved in volunteer work.

**Table 1:** Baseline characteristics (N=34).

Characteristics		Values
Age in years, median (IQR)		67 (50.0-84.0)
**Sex, n (%)**		
	Female	31 (91.2)
**Race, n (%)**		
	Asian	2 (5.9)
	Black	8 (23.5)
	White	19 (55.9)
	Multiracial	5 (14.7)
	Hispanic	1 (2.9)
**Education, n (%)**		
	High school graduate or GED^a^	2 (6.5)
	Community college or technical school	8 (25.8)
	4-year college	16 (51.6)
	Graduate school	5 (16.1)
**Marital status, n (%)**		
	Unmarried	28 (82.4)
**Employment status, n (%)**		
	Unemployed	25 (73.5)
Living alone, n (%)		21 (61.8)
History of counseling, n (%)		26 (76.5)
Depression (PHQ-9^b^), mean (SD)		11.32 (5.36)
Self-efficacy^c^, mean (SD)		24.91 (5.17)
**Quality of life indicators^d^** **, mean (SD)**		
	Social functioning	45.96 (29.63)
	Emotional well-being	50.12 (20.59)
Adaptive coping (Brief-COPE^e^), mean (SD)		51.27 (8.16)

^a^GED: General Educational Development.

^b^PHQ-9: Patient Health Questionnaire-9. Scores 0-27; 5-9 mild, 10-14 moderate, 15-19 moderately severe, 20-27 severe.

^c^Scores 10-40; higher scores indicate greater self-efficacy.

^d^Subscales range 0-100; higher scores indicate better functioning or well-being.

^e^Brief-COPE: Brief Coping Orientation to Problems Experienced. The scale has response options that range from 1 to 4 and contains 3 subscales: problem-focused coping, emotion-focused coping, and avoidant coping. A high score indicates physical or cognitive efforts to disengage from the stressor.

### Engagement and Retention

**Figure 1:**
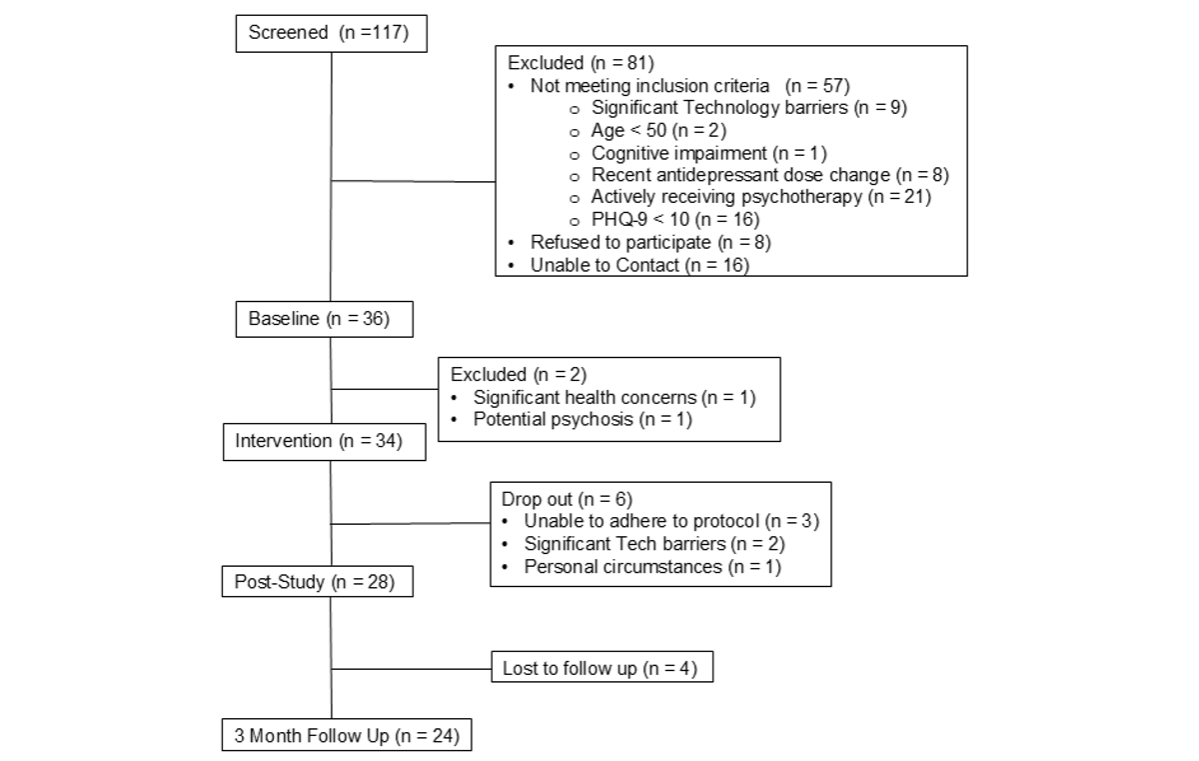
CONSORT (Consolidated Standards of Reporting Trials) diagram. PHQ-9: Patient Health Questionnaire-9.

Of the 34 participants enrolled in the study, 28 completed the intervention, and 6 dropped out (Figure 1). A total of 82% (28/34) of participants finished all 8 meetings with the PC, and 85.7% (24/28) finished follow-up assessments at 3 months. Of the 28 participants who completed the intervention, 21 were able to regularly meet with their PC through videoconferencing, while 7 had difficulties and opted to have meetings using the Zoom telephone dial-in function. Reasons for dropout included being unable to adhere to study protocol (n=3), significant technology barriers (n=2), and personal circumstances (n=1). Some participants had difficulties with technology, specifically being unable to meet through video or receive text messages. Others had a busy schedule and could not meet weekly, or had personal circumstances arise that made study participation difficult.

### Preliminary Outcomes

**Figure 2:**
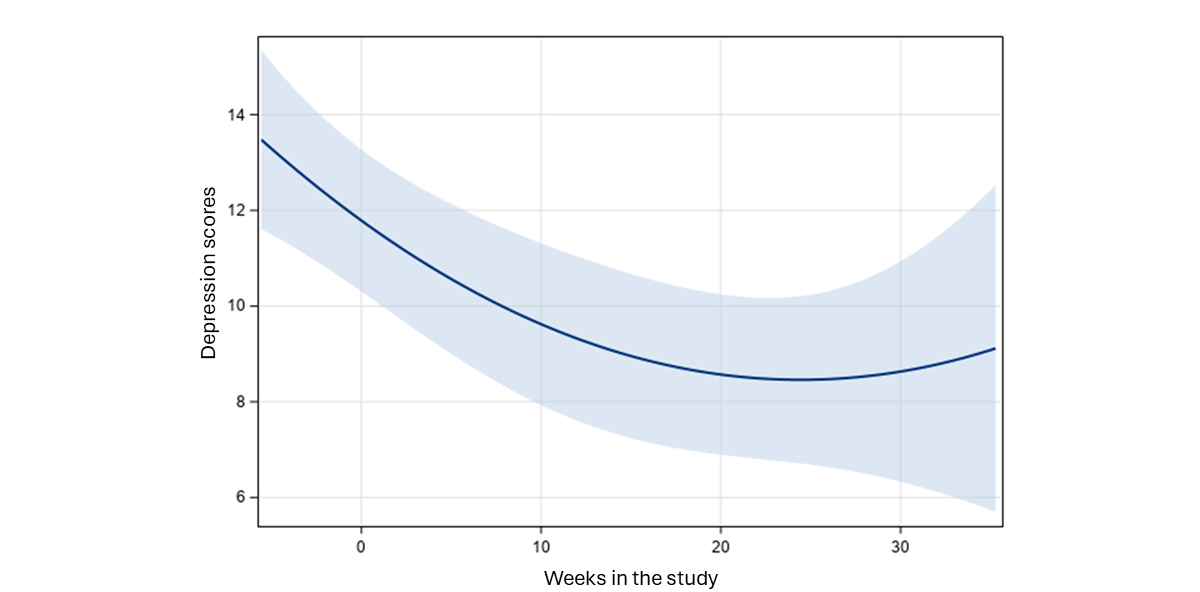
Values predicted for depression by fixed effects of the longitudinal mixed effects model. Line indicates values for depression scores (Patient Health Questionnaire-9) predicted by model fixed effects, including a borderline significant quadratic effect for weeks in the study. Colored bands indicate 95% CIs. The slight upturn in later weeks may be an artifact of the quadratic function fit.

Table 2 presents the preliminary outcomes at postintervention and 3 months. We used a mixed effects model using all available observations from enrolled participants. Depression was assessed at a minimum of 4 time points. The other outcomes were scheduled for 3 assessments; however, due to attrition and intermittent missingness, not all participants contributed data at every time point. Depressive symptoms declined over the course of the study (*F*_1, 88.8_=26.0; *P*<.001). There was also a marginally significant (*P*=.09) quadratic effect for time, suggesting that symptoms declined sharply during the intervention period, then leveled off and did not return to baseline levels (Figure 2).

**Figure 3:**
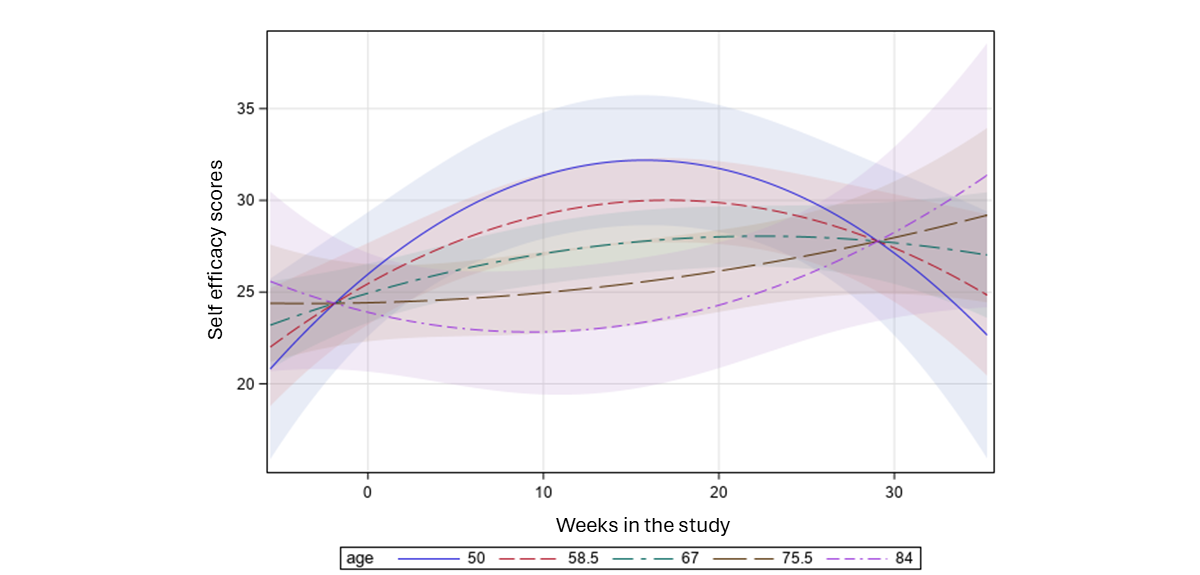
Values predicted for self-efficacy by fixed effects of the longitudinal mixed effects model, including interaction with age. Lines indicate values for self-efficacy scores (General Self-Efficacy Scale) predicted by model fixed effects, at 5 equally spaced strata of age from sample minimum (50 years) to maximum (84 years). Colored bands indicate 95% CIs.

**Figure 4:**
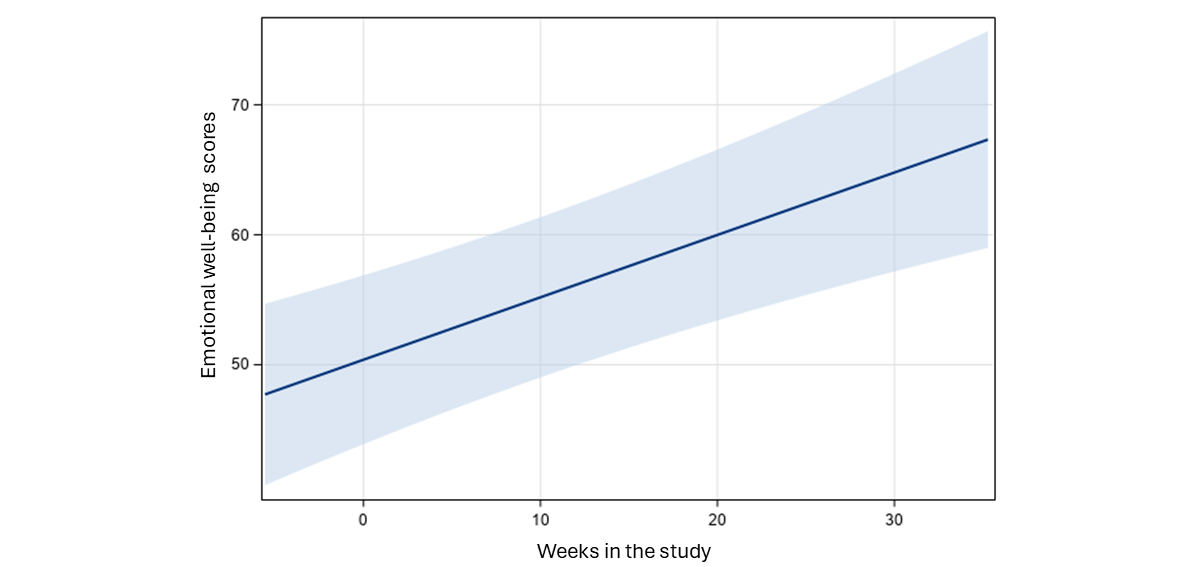
Values predicted for emotional well-being by fixed effects of the longitudinal mixed effects model. The line indicates values for emotional well-being scores (Medical Outcomes Study 36-Item Short Form Health Survey) predicted by model fixed effects. Colored bands indicate 95% CIs.

**Figure 5:**
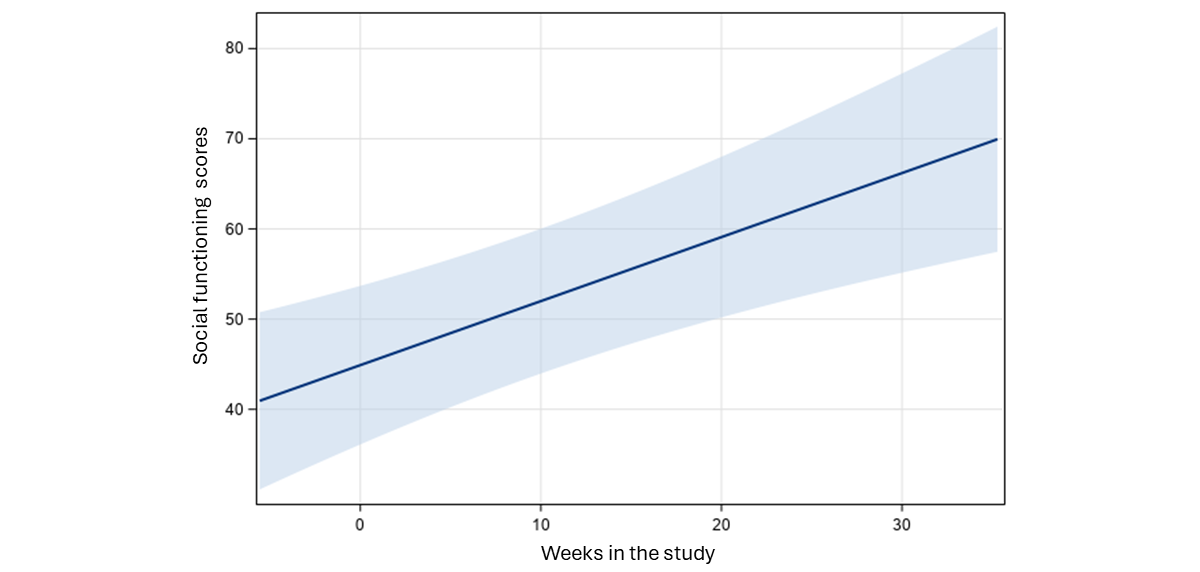
Values predicted for social functioning scores by fixed effects of the longitudinal mixed effects model. The line indicates values for social functioning scores (Medical Outcomes Study 36-Item Short Form Health Survey) predicted by model fixed effects. Colored bands indicate 95% CIs.

Self-efficacy significantly increased linearly over the intervention period (*F*_1, 50.2_=9.92; *P*=.003). A significant interaction between age and the quadratic term for time was also observed (*β*=.0011; *P*=.007), reflecting a tendency for self-efficacy to change differentially based on the age of the participant. Generally, as age increased, participants experienced a gradual increase in self-efficacy throughout the study; however, those at the lower end of the age range experienced a decrease after an initial increase (Figure 3). Emotional well-being (*F*_1, 52.9_=18.71; *P*<.001) and social functioning (*F*_1, 55_=13.94; *P*=.004) improved throughout the study (Figures 4 and 5). Effects of the intervention on adaptive coping were also observed (*F*_1, 50.4_=4.81; *P*=.033). There was a significant (*β*=.006; *P=*.049) interaction of years of education with the quadratic term for time (weeks from baseline). This effect reflected a tendency for participants with fewer years of education to increase initially and then decline again later, whereas people with more years of education tended to show more of a flat pattern across time.

The majority of participants used video chats (n=21) and a minority (n=7) dialed in to the Zoom application. Differences between phone and video chat users were assessed using a mixed effects longitudinal model with dependent variable PHQ-9, where the method of contact was used as a fixed effect predictor along with its interaction with time. This allowed the analysis to test whether the trajectory of PHQ-9 scores over time differed based on the mode of contact. The findings from this analysis found no significant differences in depression outcomes for participants using one vs the other method of contact.

**Table 2:** Results of mixed effects longitudinal analyses: values predicted for depression, self-efficacy, emotional well-being, social functioning, and adaptive coping in the longitudinal mixed effects model (N=34).

Dependent variable	Baseline, mean (SD)	Postintervention, mean (SD)	3-month follow-up, mean (SD)	*β*^a^ (95% CI)	SE	*F* test^b^ (*df*)	*P* value
Depression (PHQ-9^c^)	11.32 (5.36)	9.71 (5.59)	8.29 (4.84)	–.14 (–0.20 to –0.09)	0.03	26.00 (1, 88.80)	<.001
Self-efficacy (GSES^d^)	24.91 (5.17)	27.36 (5.50)	28.21 (3.79)	2.29 (0.83 to 3.75)	0.73	9.92 (1, 50.20)	.003
Emotional well-being (SF-36^e^)	50.12 (20.59)	55.71 (20.45)	63.83 (17.20)^f^	.48 (0.26 to 0.70)	0.11	18.71 (1, 52.90)	<.001
Social functioning (SF-36)	45.96 (29.63)	49.55 (27.53)	64.67 (22.50)^f^	.71 (0.33 to 1.09)	0.19	13.94 (1, 55)	<.001
Adaptive coping (Brief-COPE^g^)	51.27 (8.16)	55.46 (9.34)	52.57 (10.53)	2.90 (0.24 to 5.55)	1.32	4.81 (1, 50.40)	.03

^a^Unstandardized partial regression coefficient for the linear effect of time (weeks from baseline) after removal of nonsignificant quadratic term.

^b^*F* test for fixed effect of linear time.

^c^PHQ-9: Patient Health Questionnaire-9.

^d^GSES: General Self-Efficacy Scale.

^e^SF-36: Medical Outcomes Study 36-Item Short Form Health Survey.

^f^Only 23 participants provided data at the 3-month follow-up for emotional well-being and social functioning.

^g^Brief-COPE: Brief Coping Orientation to Problems Experienced.

### Fidelity

A total of 90 recordings were rated for fidelity. Nine fidelity ratings were incomplete and not included in the calculation. The scores ranged from 0 to 20, and the mean fidelity score was 17.68, with 80% of the meetings scoring above 15, indicating that the majority of meetings met the criteria for good to excellent fidelity.

### PCs’ Experience With Videoconferencing and Texting

For PCs, most technological challenges involved learning how to set up and how to conduct videoconferencing calls (Textbox 1; Q1-Q2, Q5-Q6). PCs had prior experience participating in videoconferencing meetings. However, most were not familiar with functions regarding inviting, starting, and recording meetings with participants. Continuous training, practice, and on-demand support helped mitigate videoconferencing difficulties. PCs particularly appreciated the on-demand support provided by study staff.

PCs were also often in a technology support role for the participants, which required coordination between study staff, the PC, and the participants. While PCs were learning the new technological components, they also helped their mentees navigate the same technology (Q3-Q4). Some participants had considerable difficulty using the video and audio features of videoconferencing, which meant that the PCs had to either troubleshoot or coach them through joining the meeting by phone rather than the application. PCs often had to adapt to the participants’ needs and abilities, such as modifying the level of support and using the best method of communicating directions to participants (phone call, text message, or email).

Once PCs became familiar with videoconferencing, they stated that delivering the intervention through videoconferencing was preferable to the phone if the participant was willing and able. Nonverbal cues proved invaluable for PCs, as they could gauge participants’ reactions to suggestions for coping with depression even before they were articulated (Q9-Q10). Seeing the participants over video chats also fostered a more personal connection compared to the telephone. While there were mixed feelings regarding how video chats compared to in-person meetings in connecting with the participants, PCs agreed that videoconferencing was more feasible and convenient. PCs attributed their preference for video chats to the fact that participants lived too far, and that remote delivery of the intervention was safer for themselves and participants (Q7-Q8).

**Quotations from peer coaches about their experience with video chats and texts.**

**Inexperience “hosting” meetings (challenges with using videoconferencing)**

- Q1: “...it’s no different than three-way calling, but sometimes I don’t know where it is. You’re trying to get your little cursor to go and pull it up. And where is it again?” (PC3)
- Q2: “Every now and then I got kind of off-kilter...you guys [got] me back on track. I think it was very efficient, but sometimes there were blips, as can be expected.” (PC5)

**Providing Zoom support to participants (challenges with using videoconferencing)**

- Q3: “I don’t think she’s used Zoom on her phone, so I think that’s what was hard. But then, she popped on once. I sent her the link. We tried to figure it out, but she was able to get on; I may not have seen her on screen, but she was able to listen in over the phone line.” (PC4)
- Q4: “So I would call [the participant] up and she would have to handwrite down every single step. Like one time, I gave her the meeting number. She couldn’t do that. I had to tell her to put in the meeting number and the pound sign, even though the prompt says put in the meeting number followed by the pound sign and she didn’t get that... [The participant] had technology problems. His internet had been turned off because he had not paid the bill and he got it turned on part way....” (PC6)

**Gradual familiarity with practice (challenges with using videoconferencing)**

- Q5: “I think it’s great, and I think [the technology] went very smoothly. Now, with this last mentee, not as smoothly, but when we got into a groove, we were better... But the technology, because of you and [RC], has gone leaps and bounds from the beginning of the study. You have put together such a smooth transition into the calls and submitting the wrap-up paperwork. Whereas before, we had to do it by hand and try to fax it in. So it’s much smoother, and I like working with it.” (PC5)
- Q6: “In the beginning I needed some [help]. That is true, but then once I got it I was able to do it very easily just to set up the meetings and just even to share things on the screen, to do little tricks of the trade, but in the beginning I needed-- there’s a learning curve.” (PC2)

**Preference for zoom over in-person meetings (positive experiences using videoconferencing)**

- Q7: “I don’t want to drive all over [city]. I prefer to be where I am. Plus, because of my health issues, much better, much safer for me.” (PC2)
- Q8: “With her, it was as good as an in-person meeting. But that is only because we’re both really experienced on Zoom meetings. With most of the mentees, I would say 90% of them, the in-person has it all over the Zoom...but I won’t go back to in-person because I drove one too many times. And I was driving into the city. It was a 40, 45-minute ride. I hate driving in the city. And more than once, the people just weren’t there. I will not go back to in-person. (PC6)

**Face-to-face video chats enabled more personal connection (positive experiences using videoconferencing)**

- Q9: “It’s very close to being in person, and in fact in person I might even want to wear a mask, which is a very limiting thing. I don’t have to do that. I can see her facial expressions. I can even really see the way her eyes-- her affect, so I actually-- I mean, and of course she’s in [city] and I’m in [city], so [in-person] would be impossible.” (PC2)
- Q10: “it’s really been revelatory, how much you see... if I’m explaining something...if they’re, kind of shutting down or you can tell they’re sort of on the way to, rebuffing [something], I might sort of wind it up... and check in with them right away. And if I could tell they’re really interested in it, I might, expand a little bit as I’m talking about it [something], I guess I want to say it’s information and it gives you even more of an opportunity to be serving them where they are.” (PC3)

**Apprehension toward messages being forced/ineffective (negative perspectives of text messages)**

- Q11: “Text messages were never my favorite. I found them a little forced, and maybe that’s me, and I felt like I needed to explain myself and why I was sending the messages each day. Because I don’t know if it came off as being intrusive or being “Why is she texting me every day?” (PC5)

**Determining the frequency (negative perspectives of text messages)**

- Q12: “I think it’s good, but personally I think not sending five in a week would be better. If I could send three or four instead of five, I think that’s better... I feel a text five days in a row is a bit much.” (PC4)
- Q13: “I thought it was a lot, too much, and maybe paring it down to three times a week.” (PC5)

**Strengthening the bond between peer and participant (positive experiences of sending text messages)**

- Q14: “I love the text. It’s a way to underline, encourage, ... you’re not alone… just hi, here’s your day. Hopefully it’s a little bit brighter or more insightful or something because you’re part of Peers+.” (PC3)
- Q15: “She’s someone who likes to respond, so she’ll respond to anything and she would want to send me something. Like she would be open to sending me a picture or something that happened to her.” (PC4)

**Positive reinforcement for participants (positive experiences of sending text messages)**

- Q16: “I was very skeptical. I was like thinking, well, that would overload someone, or it would turn me off if somebody wrote me every day like that, but she really loved it, and she felt very connected to me, and she felt very instructed or encouraged or inspired by me, so therefore it was a fantastic thing, I thought.” (PC2)
- Q17: “Effective is when people were accepting of them [texts], sometimes they would answer me. I would send a happy message, and they would say “Thank you very much.” (PC5)

When asked about their perceptions of the added texting to the intervention, PCs initially provided mixed responses (Textbox 2). Some embraced the text messages immediately as a way to further encourage and connect with participants outside of meetings (Q14-Q15). Others reacted with more hesitation, unsure of whether mentees would like and how they would respond to the text messages (Q11, Q16-Q17). One PC acknowledged that she was initially skeptical of supportive texts; however, when she observed the positive responses by the participants, she understood the value of text messages (Q11).

**Quotations from participants about their experience with video chats and texts.**

**Unfamiliarity with Zoom (challenges with using videoconferencing platform)**

- Q1: “I had to have a lot of support with [Zoom] because I didn’t understand it. I didn’t know how to do it.” (P15)
- Q2: “The only thing we didn’t do that they did request was a Zoom call because I wasn’t prepared for that and I didn’t know how to do it. But I regret not doing it because it’s funny talking to someone that long and not knowing what they look like.” (P16)

**Technical problems (challenges with using videoconferencing platform)**

- Q3: “The sound, I don’t know why sometimes my sound wasn’t working, but whatever.” (P07)
- Q4: “...she was trying to tell me what to do [to connect on Zoom] and sometimes it would work. This week we would do it and she’d tell me to do it and it would work; the next week she’d tell me to do it and it didn’t work. And so I think we probably out of eight sessions, maybe two sessions we had where we could literally see each other and hear each other.” (P21)

**Embracing Zoom despite initial skepticism (challenges with using videoconferencing platform)**

- Q5: “Maybe, I never really believed in online, this type of thing... Maybe initially I felt that way, but that stigma that I had in my head was long gone.” (P07)
- Q6: “I had a little bit of a problem back and forth when I used my tablet. And the more I did it, the better it was. I did have a little bit of struggles with it...every once in a while on my tablet, it wasn’t quite as easy. But once I got a hang of them, it was good.” (P26)

**Lack of privacy (challenges with using videoconferencing platform)**

- Q7: “I don’t like the way I look on video. I actually did it by accident one night, I put myself on video call to my daughter-in-law and I said, oh my god, I was shocked.” (P16)
- Q8: “Don’t want you to see that. I just don’t like it. I think it’s kind of an invasion. Unless it’s your mate, your husband, your girlfriend, boyfriend, I don’t want everybody to see me.” (P28)

**Inferior compared to In-person meetings (challenges with using videoconferencing platform)**

- Q9: “...to replace an in-person interaction, I think it’s okay... having interactions with people in person, it’s just more personable. You’re able to see the body language a little bit better. Sometimes it kind of drops walls. I think that in-person interactions, you are probably able to make a connection faster than [when] you are online.” (P27)
- Q10: “[Zoom] meant that I didn’t have to go anywhere, that I didn’t have to dress up or do this or do that...And we had been coming off of zooming for those couple of years when we were all locked down with COVID...any way you look at it, I’d much prefer to meet in person directly with another human being, always. However, this was a good thing. It was fine.” (P33)

**Convenience (benefits of using Zoom)**

- Q11: “The Zoom is convenient, you’re in the comfort of your home... you can be in pajamas, and you can be feeling more comfort than being outside in a building” (P02)
- Q12: “I feel like the anxiety of people going, getting to where they’re going. You don’t know that the traffic, the stress of what they’re experiencing before that arrival. And then the person they’re going to meet with, you don’t know if they’re rushing from one meeting to rush to you. And so, I feel like everyone was comfortable in their own space.” (P24)

**Personal Connection (benefits of using Zoom)**

- Q13: “I think the Zoom call is much better than over the phone because you can actually see the person, and I think that makes a very big difference. It’s just a warmer feeling, I think.” (P04)
- Q14: “just to talk to someone face to face makes it more, ... like she has more passion when I can see [her] face and what she says to me it’s more of a personal relationship over Zoom” (P02)

**General Dislike of Text Messages (negative experiences with text messages)**

- Q15: “I hated the texting. Hated it. Hated it. And I don’t know why I hated it so much, but I just did...there’s just something that feels very intrusive about that.” (P29)
- Q16: “I’d like to talk instead. I think text messages can be very impersonal and they should be done only if you [are close].” (P16)

**Effect on mood (positive experiences with text messages)**

- Q17: “[Text messages] definitely lightened my day, my mood, the way I was feeling, because some days I was down. She’d send this text messages and I would just smile knowing it was her thinking about me and that helped relieve some of my tension.” (P2)
- Q18: “I’ll see a little message from her. “Don’t get discouraged. You’re doing good. Rome wasn’t built in a day.” ...I felt like almost I knew her. She could be a friend of mine who was trying to help me.” (P32)

**Reinforcement of goals (positive experiences with text messages)**

- Q19: “The text messages reinforced what we were trying to accomplish, meaning just giving me that extra support, that extra push that she thought I needed to get me through.” (P9)
- Q20: “Some of her text messages were just motivation, reminding [me of] the work-- I have everything I need to be successful. She would remind me that at least once a week, and just wishing me good days or wishing me a happy, weekend-- things like that.” (P34)

**Importance of personalizing text messages (positive experiences with text messages)**

- Q21: “I’m not religious, I appreciated that there were no religious references. I’m sure, of course, someone who’s religious might appreciate a mention of things in accordance with their faith.” (P07)
- Q22: “She sent me Thoreau quotes and things we talked about in session, which I loved. She sent me little things... just to remember to stop and breathe and take some time and just be gentle.” (P29)

### Participants’ Experience With Videoconferencing and Texting

Similar to the PCs, participants who successfully met over video described how seeing their PC facilitated a personal connection despite technical issues (Textbox 2) and video afforded them the convenience to attend meetings reliably (Q11-Q12). One participant described how their initial skepticism toward remote sessions faded as they continued the intervention (Q5-Q6). Most participants agreed that while video chats were an acceptable format and conferred advantages, they could not replicate the level of connection that an in-person session would provide (Q9-Q10). Participants who declined to meet over video described how it felt invasive to do so in their homes and said that they disliked seeing themselves on camera and feared judgment by the PC about the appearance of their person or their homes (Q7-Q8).

Participants reported a range of attitudes in response to receiving text messages. Participants who had regular commitments (eg, work) preferred fewer texts, and 1 participant reported an aversion to texts (Q15). However, most participants expressed positive feelings toward the text messages. Text messages reinforced the trusting relationship between PCs and mentees and provided participants with another form of connection and engagement within the intervention (Q17-Q18). As intended, participants felt that the text messages reinforced skills and coping strategies that were discussed during meetings and added a layer of accountability (Q20).

## Discussion

### Principal Findings

Our study demonstrated the feasibility and acceptability of an individual peer support intervention that focused on increasing depression coping and delivered remotely by older adult PCs, using video chats and enhanced by unidirectional text messages. Technology delivery was feasible and well accepted by both participants and PCs, with adequate training and on-demand support. Preliminary outcomes for depression, emotional well-being, self-efficacy, and social functioning showed positive results and supported the need for future effectiveness testing in an RCT.

Engagement in the intervention was high, with 82% (28/34) completing all 8 weeks of the meetings. Research has shown that peer support is an engaging intervention in community settings and is commonly used for those who underuse traditional health services [49,50]. Additionally, older adults desire to speak with someone with whom they can identify, such as someone who is older and can understand the struggles of their shared health condition [41]. Furthermore, the isolating experience of depression and aging itself may contribute to the appeal of a psychosocial intervention that is delivered by lay persons, without the stigma of engaging in professional care. Finally, although video chats were not considered as a substitute for in-person meetings, video chats were perceived to offer sufficient advantages, such as convenience and safety, that contributed to feasibility and high engagement.

Our findings are consistent with previous studies demonstrating the effectiveness of various technological modalities to deliver mental health services that show benefit [24,41,51]. Telehealth is used to deliver psychotherapy routinely and has been shown in numerous studies to be effective for mental health outcomes, when compared to delivery in person or by telephone [30,52-54]. In the area of peer support, pilot studies have demonstrated the feasibility of using online and technology-enhanced peer support to increase reach and access [29,55,56], and online support groups have been shown to increase life satisfaction and positive interactions [57]. However, no RCTs of technology-enhanced peer support have been done in the United States [58-61]. There is a great need to develop technology-enhanced interventions for mental health for older adults that are engaging and accessible and incorporate aspects of the user, intervention characteristics, and the availability of technology support to increase intervention engagement and usage [62].

Use of texting for the older adult population is less common but emerging. Older adults are engaging more with texting than in previous years, and the use of texting has been shown to be effective in promoting health and well-being in older adults in rural and urban areas in international settings [63-65]. One texting intervention successfully decreased loneliness among older adults [66] with low-intensity cognitive behavioral therapy [65]. Texting was a feature of a self-management digital application used by peer specialists to promote empowerment and self-efficacy that was shown to be feasible [29].

Although video chats and texts can be used as independent interventions and are studied as such [55], they were combined as complementary modalities in our study. Video chats enabled psychosocial intervention through complex processes of social learning and social support. Simple, unidirectional texts served as adjuncts, reinforcing the social connection between the video chats, providing added encouragement to use coping strategies, and sustaining accountability. This is consistent with prior work using texting to support psychosocial interventions [40-42]. While text messaging has shown promise as a tool for promoting behavior change—such as smoking cessation or increased physical activity—addressing depression and social isolation may require something that texts alone cannot provide: a sense of genuine relationship and trusted human connection. For these deeper psychosocial needs, a blended approach that combines text with other modalities, such as phone calls, video, or in-person contact, may prove more effective than any single modality alone [67].

Our study contributes to the knowledge regarding technology integration in peer support interventions and advances our understanding of the support needed to make video chats and texting engaging and accessible. Older adults in our study were curious and willing to learn, and comfort was achieved with practice. However, this occurred when technology support was provided in ways that were accessible. We designed the technological interface for simplicity of use. We considered the uneven access to devices, comfort, and skills with Zoom and text, and tailored the training for PCs and older adult participants. The study team also offered on-demand support, which was important for participants specifically, compared to the PCs, who offered technological support to the participants as needed.

The perceived advantages of telehealth and the increasing digitalization of health care services require enabling older adults to use technology by providing knowledge and addressing differential access to devices. One study found that among older adults, age and income were not absolute barriers if technology-enabling factors were in place [68]. Additionally, successful applications of technology, in addition to improving outcomes, have the potential to change attitudes toward technology and increase technology self-efficacy [69]. This will require interventions that are simple and engaging, integrating user-centered design thinking, and incorporating older adults in the development process [70].

The use of technological innovations to deliver health services will continue; however, older adults in our study expressed the superiority of in-person services [71]. This prompts us to consider the implications of the modalities for communication that are used in psychosocial interventions, especially when the social connection, the quality of the relationship, and trust building are important, such as in peer support [72]. As previous studies have shown and our study has found, there are specific advantages and disadvantages of video vs in-person communication, and these may have to be weighed with the goals and characteristics of the intervention, the target population, and the interventionists.

### Strengths, Limitations, and Future Directions

We note several strengths of our study. We were successful in recruiting a group with a range of ethnic identities. Approximately half of the participants identified as individuals of color or mixed race, who are often underrepresented in psychosocial research studies. The use of qualitative interviews allowed us to capture the detailed perspectives of participants and PCs, revealing the benefits and challenges of videoconferencing and texting. However, our study also has limitations that should be considered. This study was a feasibility trial with a small sample size (N=34), so our results cannot be generalized. Our sample was majority women and college-educated, which may confer increased access to digital technology and text messages. Our understanding of the intervention’s feasibility among individuals with lower education status is limited, but it did provide insight into adaptive coping when added to the model as a covariate. We were also unable to interview the participants who dropped out due to significant technological challenges. Future feasibility studies of technology-delivered interventions among men and persons with lower educational status are needed, as well as exploration of recruitment and retention methods when technology proves to be a barrier to participation. Lastly, this was a single-arm study, so a comparison of treatment effects vs any possible spontaneous change that might have occurred in a no-treatment control group could not be ascertained. The preliminary outcomes generate hypotheses that need to be tested in an RCT.

### Implications

Peer support interventions are accessible, community-based interventions with significant potential to engage older adults and address barriers to depression care that are intractable and can lead to poor outcomes. Older adult volunteers or peer specialists who are credentialed in the majority of states in the United States are an existing workforce that can be leveraged to provide public health approaches to depression care. Regarding integration of technology in intervention design, older adults frequently face technology literacy and access challenges, yet are motivated to learn and increasingly rely on technology in their daily lives. Tech support for both participants and PCs should be a core element of intervention design to improve feasibility and implementation success.

## Abbreviations

PC: peer coach
PHQ-9: Patient Health Questionnaire-9
RCT: randomized controlled trial
REDCap: Research Electronic Data Capture

## Data Availability

The datasets generated or analyzed during this study are available in the National Institute of Mental Health Data Archive. Qualitative data are not publicly available due to participant privacy and confidentiality concerns but are available from the corresponding author on reasonable request.
